# Factors That Affect the False-Negative Outcomes of Fine-Needle Aspiration Biopsy in Thyroid Nodules

**DOI:** 10.1155/2013/126084

**Published:** 2013-06-27

**Authors:** Orhan Agcaoglu, Nihat Aksakal, Beyza Ozcinar, Inanc S. Sarici, Gulcin Ercan, Meltem Kucukyilmaz, Fatih Yanar, Ibrahim A. Ozemir, Berkay Kilic, Kasim Caglayan, Dilek Yilmazbayhan, Artur Salmaslioglu, Halim Issever, Selcuk Ozarmagan, Yesim Erbil

**Affiliations:** ^1^Department of General Surgery, Istanbul Medical School, Istanbul University, 34493 Capa, Istanbul, Turkey; ^2^General Surgery Clinic, Bagcilar Research and Training Hospital, Istanbul, Turkey; ^3^Department of Pathology, Istanbul Medical School, Istanbul University, Istanbul, Turkey; ^4^Department of Radiology, Istanbul Medical School, Istanbul University, Istanbul, Turkey; ^5^Department of Public Health, Istanbul Medical School, Istanbul University, Istanbul, Turkey

## Abstract

*Background*. The purpose of this study was to assess the factors that affect the false-negative outcomes of fine-needle aspiration biopsies (FNABs) in thyroid nodules. *Methods*. Thyroid nodules that underwent FNAB and surgery between August 2005 and January 2012 were analyzed. FNABs were taken from the suspicious nodules regardless of nodule size. *Results*. Nodules were analyzed in 2 different groups: Group 1 was the false-negatives (*n* = 81) and Group 2 was the remaining true-positives, true-negatives, and false-positives (*n* = 649). A cytopathologist attended in 559 (77%) of FNAB procedures. There was a positive correlation between the nodule size and false-negative rates, and the absence of an interpreting cytopathologist for the examination of the FNAB procedure was the most significant parameter with a 76-fold increased risk of false-negative results. *Conclusion*. The contribution of cytopathologists extends the time of the procedure, and this could be a difficult practice in centres with high patient turnovers. We currently request the contribution of a cytopathologist for selected patients whom should be followed up without surgery.

## 1. Introduction

The prevalence of thyroid nodules in the general population is estimated to be 4–7% [1], and clinicians who evaluate these patients must make a decision to perform surgery or only follow up with reexaminations. In the diagnosis and management of thyroid nodules, fine-needle aspiration biopsy (FNAB) has been widely accepted as the most accurate test [2–5]. The routine use of FNAB has reduced the number of unnecessary surgical procedures and increased the detection of thyroid cancer at earlier stages [6].

It has been shown that the support of ultrasonography (USG) increases the sensitivity, specificity, and success rate of FNAB [7]. When a positive result for malignancy is obtained, the predictive value of the test is almost 100%. On the other hand, there is a marked decrease of this value with the presence of a negative result [3]. Thus, there is a debate in the literature regarding the role of FNAB of large nodules in the diagnosis. Some studies report high false negative results [8, 9], whereas others note that USG-guided FNAB is accurate regardless of nodule size [10].

Although there are studies regarding the accuracy of FNAB in the literature, the aim of this study was to evaluate the factors that affect the false-negative outcomes of FNAB.

## 2. Materials and Methods

We retrospectively reviewed the medical records of patients who underwent FNAB procedure and then underwent thyroid surgery in our clinic between August 2005 and January 2012. 

The cytopathologic diagnosis of thyroid FNAB was assessed in four categories including malignant, indeterminate (suspicious for malignancy), benign, and nondiagnostic. Malignant results included atypical follicular cells with malignant nuclear features such as papillary, follicular, medullary, and undifferentiated thyroid carcinomas. Specimens were categorized as suspicious when cytology included cytological atypia, pleomorphic and abnormal nuclei, but did not fulfill the criteria for malignancy. Benign cytology indicates colloidal nodules, nodular hyperplasia, lymphocytic thyroiditis, and Graves' disease. The nondiagnostic group included samples with a minimum of six groups of well-visualized (i.e., well-stained, undistorted, and unobstructed) follicular cells, with at least ten cells per group visualized in at least two smears. We excluded the nondiagnostic group (*n* = 161) from the study and analyzed the remaining 3 categories. The remaining 730 lesions were included in this study, and the final sample consisted of 590 patients.

Our criteria for performing surgery on thyroid nodules were malignant or suspicious for malignancy results from FNAB, nodule size of >4 cm in diameter, and increase in nodule volume >50% between two followups and patient's desire for surgery.

The numbers of true-negative (TN), true-positive (TP), false-negative (FN), and false-positive (FP) results were calculated. The malignant and indeterminate FNABs were considered to be true-positives (TP) in cases where histological examination revealed a malignancy, but were considered to be false-positives (FP) when no malignancy was found. The benign FNAB was considered to be true-negative (TN) when the histological finding was benign and false-negative (FN) for cases of histologically proven malignancy. The diagnostic accuracy, sensitivity, specificity, positive predictive value (PPV), and negative predictive value (NPV) of USG-FNABs were calculated. Sensitivity, specificity, and accuracy were determined according to the following formulae:

- PPV (%) = TP/(TP + FP) × 100,
- NPV (%) = TN/(TN + FN) × 100,
- Sensitivity (%) = TP/(TP + FN) × 100,
- Specificity (%) = TN/(TN + FP) × 100,
- Accuracy (%) = TP + TN/(TP + TN + FP + FN) × 100.

In patients with multinodular goiter, FNAB analysis was undertaken from the suspicious nodule regardless of nodule size. Clinical data included preoperative USG imaging, FNAB cytology, and final pathology. All FNAB procedures were performed using ultrasonographic guidance and with or without cytopathologists. All nodules had undergone thyroid surgery according to our previously mentioned surgical criterias. Finally, FNAB cytological outcomes were compared with the results of postoperative final pathological results.

### 2.1. FNAB Procedure

All FNAB procedures were carried-out under ultrasonography guidance and were performed by a radiology specialist using a broadband linear transducer (VFX 13-5, Sonoline Antares, Siemens, Germany). Analyses were taken from the suspected malignant nodules when USG indicated suspicious findings (i.e., a hypoechoic nodule in association with punctuate calcifications and/or irregular borders) regardless of nodule size in multinodular glands. FNAB was performed under direct USG visualization with a 23-gauge needle attached to a 5 mL disposable syringe depending on the preference of the radiologist. During the procedure, the patient was kept in the supine position with a slight hyperextension of the neck. Local anesthesia was not routinely used for procedures. For obtaining a sufficient amount of diagnostic material, two or three aspirations were usually performed. After aspiration, the obtained samples were smeared, fixed on glass slides, and stained. One to three slides from each of the patients were stained with Papanicolaou stain and Giemsa stain to confirm the presence of thyroid follicular cells. Each mass was described with regard to the imaging features including nodular status (solitary, multinodular), nodule size, echogenicity, and internal components. Internal components were calculated in 3 subgroups such as solid, cystic, and mixed. The needle was introduced toward the solid portion of mixed nodules. Cytological examination of FNAB specimens was performed by experienced endocrine cytopathologists.

### 2.2. Statistical Analysis

Data were analyzed using SPSS 19.0 for Windows. Results were expressed as mean ± SD. Comparisons of data were done by *t*-test, chi-squared test, correlation analysis, and logistic regression analysis. Results were considered statistically significant when the two-tailed *P* value was less than 0.05. 

## 3. Results

### 3.1. General Characteristics

There were 730 nodules analyzed in our study. The results included true-positives (*n* = 239), true-negatives (*n* = 365), false-positives (*n* = 45), and false-negatives (*n* = 81). Nodules were analyzed in 2 different groups according to the results of the FNAB: Group 1 was false-negatives (*n* = 81) and Group 2 was the remaining true-positives, true-negatives, and false-positives (*n* = 649). Cytopathologist attended in 559 (77%) of FNAB procedures. Internal components of nodules were solid in 54% (*n* = 396) and mixed in the remaining 46% (*n* = 334) (Table 1).

### 3.2. Groups Characteristics

Group 1 had 81 FN nodules and group 2 had 649 TN, TP, and FP nodules. The mean nodule size was 28.5 mm (±15.5) in Group 1 and 24.0 mm (±15.0) in Group 2. The procedural attendance of cytopathologists in Group 1 and Group 2 were 6 (7.4%) and 553 (85.2%), respectively. Group 1 had 42% solid nodules and 58% mixed nodules, whereas Group 2 had 56% solid nodules and 44% mixed nodules. There were no nodule size and internal component differences between the two study groups (Table 2). 

### 3.3. Correlation Analysis

There was a positive correlation between the nodule size and false negative results (*r*_*s*_ = 0.093, *P* = 0.012), whereas there was a statistically significant negative correlation between the attendance of cytopathologist and false-negative results (*r*_*s*_ = −0.577, *P* < 0.001). The analyses showed that there were no significant correlations between the internal components of the nodule and false-negative results, but the attendance of a cytopathologist was the most significant parameter which correlated with false-negative results for FNAB.

### 3.4. Logistic Regression Analysis

The results of the logistic regression analysis for false-negative results are given in Table 3. Nodule size and cytopathologists were independent significant variables of the false-negative results for FNAB.

### 3.5. False-Negative Rates of Subgroups according to Nodule Size

In the analysis, we also created subgroups to calculate the false-negative ratio according to nodule diameter. Nodule size of 2-3 cm had a 4.9-fold increase (OR: 4.9; 95% CI: 1.4–16.8), nodule size of 3-4 cm had a 5.0-fold increase (OR: 5.0; 95% CI: 1.3–18.6) and no attendance of cytopathologists had a 76-fold (OR: 76; 95% CI: 31.8–181.5) increased risk for false-negative results (Table 3). 

## 4. Discussion

There are numerous reports in the literature regarding larger thyroid nodules having a higher rate of malignancy, and this has inclined the recommendation of thyroidectomy for all nodules greater than 3 cm [9, 11, 12]. Due to this recommendation, the utility of FNAB has been gaining importance as a diagnostic aid in the management of thyroid nodules. In one of these studies, Kuru et al. stated that the false-negative rate of FNAB is low for thyroid nodules <4 cm and also stated that it was not the nodule size but the size of papillary thyroid carcinoma in the nodules that was more important. In another study, Siddiqui et al. also demonstrated that papillary thyroid carcinomas in the FNAB diagnostic group were significantly larger than in the missed group.

When we reviewed the literature, we found 4 large studies that had considered the performance of ultrasound-guided FNABs in large thyroid nodules. Half of these studies had reported false-negative rate of 0.7 to 1.8% [10, 13], whereas the remaining two studies had a rate of 9–16% [8, 14]. Our false-negative result for FNAB was 11%.

Yoon et al. [13] reported a large series of 661 FNAB procedures and noted that it was an accurate diagnostic tool that could be used regardless of size when it was performed under ultrasonography, reporting a false-negative rate of 1.8%. They also mentioned that the key points of success were the visualization of the needle tip with ultrasound during aspiration which helps in reducing the sampling errors and improves the accuracy of FNAB regardless of lesion size and that the contribution of a cytopathologist decreases the false-negative results in addition. Porterfield et al. [10] also noted the same outcomes and the same causes of false-negative outcomes such as inaccurate sampling and mistaken cytology analysis.

In contrast to these studies, McCoy et al. [8] reported on 223 FNAB procedures and found a false-negative rate of 13–16%. In this study, they analyzed clinical data from 223 patients, and false-negative FNAB results were reviewed by their cytopathologist. They noted that benign FNAB cytology of large thyroid nodules has a high false-negative rate for cancer at up to 13% and missed follicular lesions up to 34%. Also in their review, they suggested that an experienced cytopathologist could eliminate most of the false-negative results at the outset.

Our results were very similar to this last study with a false-negative rate of 11%. In our study, there was a positive correlation between the nodule size and false negative results, whereas there was a statistically significant negative correlation between the contribution of a cytopathologist and false-negative results. Parallel to the literature, nodule size was one of the independent factors in our study.

In comparison to smaller nodules, the false-negative rate of FNAB seems to be higher for malignancy in larger nodules, especially in nodules measuring larger than 4 cm [4, 8]. As a consequence of this, there are several studies regarding the efficacy of FNAB and management of large thyroid nodules; such lesions should be resected regardless of the FNAB result [9, 15]. Some of these reports conclude that large nodules lead to increased risk for malignancy [4, 16, 17], whereas others note the contrary [18, 19]. 

As previously outlined by other authors, FNABs sometimes do not reflect the histology of the entire nodule [10, 20]. This could be due to inaccurate sampling, dependency on a skilled USG operator and interpreting cytopathologist as Gharib and Goellner [3] mentioned previously in one of their studies. In a recent study, Pinchot et al. [4] reported that FNABs for thyroid nodules larger than 4 cm have inaccurate results and need diagnostic lobectomy; however, we believe that their study had been affected by factors that influenced their outcomes. These included, being without ultrasound guidance necessitating a free-hand biopsy, attendance of a cytopathologist was not routine and they did not analyze the contribution of the cytopathologist in 2 comparable groups, and the experience of the cytopathologist was not reported. In a prospective study of 159 patients, Carrillo et al. [21] paid attention to the nodule size and concluded that the only factor related with false-negative FNAB results was nodules of 4 cm or larger.

In our study, the most significant factor was the contribution of the cytopathologist. In the absence of a cytopathologist, there was a 76-fold increased risk of false-negative results; however, the wide CI values decrease the significance of reliability. In our opinion, the contribution of a cytopathologist requires additional time for the procedure; and it could be difficult to perform FNAB with cytopathologists routinely especially in clinics which have high patient turnovers. However, we suggest that due to the significant correlation of false-negative outcomes, the contribution of a cytopathologist should be used especially for the lesions that require followup.

In conclusion, this is one of the largest series in the literature, and we wish to highlight the importance of the contribution of a cytopathologist for FNAB procedures to reduce the high false-negative results for malignancy in thyroid nodules. FNAB reliability can be affected by several factors including sampling errors, suboptimal slide preparations, and the requirement for experienced radiologist and cytopathologists. Although increasing nodule size was associated with increased false-negative results, we believe that using the combination of an experienced ultrasound operator and an interpreting cytopathologist can significantly decrease false-negative results.

## Figures and Tables

**Table 1:** General characteristics of the nodules.

Parameters	Number of nodules
Pathology	
Malignant	412
Benign	318
Cytopathologist	
Included	559
Not included	171
Nodule internal components	
Solid	396
Mix	334

**Table 2:** Comparison of study groups.

Parameters	Group 1 (*n* = 81)	Group 2 (*n* = 649)	*P*
Nodule size (mm) Mean (±SD)	28.5 ± 15.5	24.0 ± 15.0	NS
Contribution of cytopathologist			<0.001
Yes	6	553	
No	75	96	
Nodule internal components			NS
Solid	34	362
Mix	47	287

**Table 3:** Logistic regression analysis results regarding to the false-negative rate.

Independent variables	Dependent variable	*P*	OR	95% CI
Nodule size (2-3) cm	FN	0.012	4.9	1.4–16.8
Nodule size (3-4) cm		0.018	5.0	1.3–18.6
No attendance of cytopathologists		<0.001	76.0	31.8–181.5

## Abbreviations

FN: False negative
FNAB: Fine-needle aspiration biopsy
FP: False-positive
NPV: Negative-predictive value
PPV: Positive predictive value
TN: True-negative
TP: True-positive
USG: Ultrasonography.
